# Evolution of Surface Morphology of Spin-Coated Poly(Methyl Methacrylate) Thin Films

**DOI:** 10.3390/polym13132184

**Published:** 2021-06-30

**Authors:** Navid Chapman, Mingyu Chapman, William B. Euler

**Affiliations:** Department of Chemistry, University of Rhode Island, 140 Flagg Road, Kingston, RI 02881, USA; navid_chapman@uri.edu (N.C.); mingyu_chapman@uri.edu (M.C.)

**Keywords:** PMMA, polymer wrinkles, Rayleigh–Bénard–Marangoni convection

## Abstract

The morphology of sub-micron poly(methyl methacrylate) films coated to glass supports by spin coating from toluene is examined using surface profilometry. Wrinkled surfaces with local quasi-sinusoidal periodicity were seen on the surfaces of films with thicknesses of larger than 75 nm. The surface wrinkles had large aspect ratios with wavelengths in the tens of microns and amplitudes in the tens of nanometers. Wrinkles that formed during spin-coating are attributed to surface perturbations caused by Rayleigh–Bénard–Marangoni convective instabilities. The effects of film thickness, coating solution concentration, and drying rate on the thin film surface morphology are investigated. The results can be used to prepare surfaces with controlled morphology, either smooth or with periodic wrinkles.

## 1. Introduction

We have previously reported that xanthene dyes are effective sensors for explosives [1,2,3]. The key discovery was that significant fluorescence signal enhancement can be achieved using a three-layered sensor design comprised of a substrate, a spin-coated sub-micron-layer of a transparent polymer, and topped with the fluorophore monolayer [2]. Further, we showed that the nature of the polymer layer, both in terms of thickness and morphology, influenced the sensor performance [3]. This led us to undertake fundamental research on the thin film formation dynamics of the polymer layer to better understand the sensor characteristics. This will allow the preparation of surfaces with predefined structures to examine the optimum morphology for sensor fabrication or other applications. For those applications where a smooth surface is desired, the conditions required to avoid wrinkles are equally important.

Spin-coating is a favored technique for forming uniform films with flat surfaces, which offer several performance advantages regarding electronic properties and device stability [4,5]. In the spin-coating technique, the coating material is dissolved in a volatile solvent, the substrate is fixed to a rotor, a volume of coating solution is deposited on the substrate. As the rotor is set into motion, the fluid spreads until resisted by viscous drag, then evaporation drives fluid thinning in the final coating stages. Spin-coating is a dynamic process in which evaporation can drive fluid instabilities to roughen a fluid surface. It has been reported that the wrinkled surfaces found on spin-cast poly(methyl methacrylate) (PMMA) thin films are vitrified interfacial flow cells driven by gravity and surface tension phenomena. Individual convective cells self-arrange into a semi-organized pattern that resembles a sunflower, with convection rolls radiating from a central cluster of flow cells that share some resemblance to the hexagonal flow cells that Henri Bénard observed with whale oil [6]. Self-assembled patterned surfaces are taken advantage of in many thin film applications, such as microfluidics, stretchable and foldable electronics, and advanced sensing arrays of medical diagnostic equipment [7]. 

Evaporative cooling at the surface of the spin-coating droplet leads to the development of a temperature-concentration gradient. When solvent evaporation is greater than condensation, non-equilibrium conditions drive interfacial fluid motion. Convective flow can substantially enhance heat and mass transfer during film drying. Surface wrinkling instabilities evolve when tension experienced at the interface exceeds the resistance to deformation. Because the polymer has a higher glass transition in solution, polymer enrichment during film drying brings about a rapid glass transition and mobility is suppressed. The polymer packing arrangement becomes fixed and relaxation to thermodynamic equilibrium conformations is restricted [8]. 

**Scheme 1:**
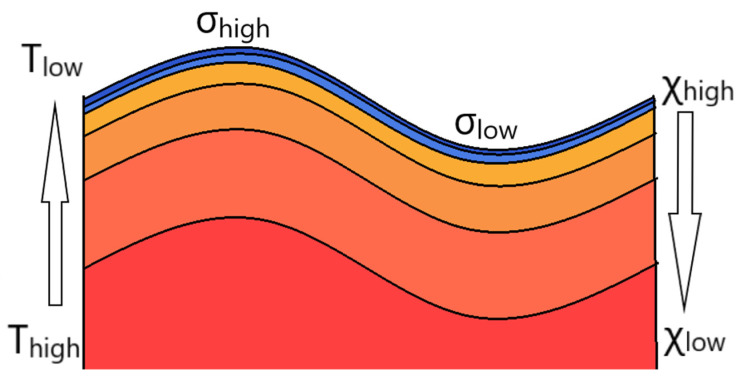
Schematic of temperature-concentration gradient on volatile fluid droplets. *T* is temperature, σ is surface tension, and χ is polymer mass fraction. Solvent evaporation causes localized cooling at the surface of the spin-coating polymer fluid. Convective instabilities can roughen the surface to decrease surface energy.

A basic description of convective flows lays the framework for the theoretical experiment. In natural convection, also known as Rayleigh convection, gravitational effects are influential and buoyancy forces dominate. Buoyancy force is associated with the dimensionless Rayleigh number (*Ra*):

(1) $$Ra=\frac{\alpha gh^{3}\Delta T}{\kappa \nu }$$

where *a* is an expansion coefficient, *g* is the acceleration due to gravity, *h* is the vertical thickness of the fluid layer, D*T* is the temperature gradient between the substrate and surface, *k* is the thermal diffusivity, and *n* is kinematic viscosity. Because density typically varies inversely with temperature, evaporative cooling brings about a vertical temperature-concentration gradient in which a heavier fluid lies above a lighter fluid as shown in Scheme 1. Gravity can induce heat and mass transport, which will continue until a critical point where stability between thermal diffusion and buoyancy convection exists. The downflow of cooler condensed particles drives the warmer, less dense particles upwards and starts circular fluid convection [9,10].

In thermocapillary convection, also known as Marangoni convection, surface tension phenomena are influential. The dimensionless Marangoni number (*Ma*) describes the rate of thermal transport by diffusion in relation to surface tension driven convection [11]:

(2) $$Ma=-\frac{\partial \sigma }{\partial T}\frac{h\Delta T}{\kappa \eta }$$

where $\frac{\partial \sigma }{\partial T}$ is the rate of change of the surface tension with respect to temperature and *h* is the dynamic viscosity. In a similar manner to density, surface tension is partly a function of concentration and temperature. Evaporative cooling creates a localized temperature-concentration gradient at the free interface. When the surface tension of the solvent is less than the solute, solvent evaporation will lead to an increase in interfacial free energy. Surface tension driven convective instabilities are known as the Marangoni effect. In thermocapillary convection, surface tension responds to changes in heat and density. Heat and mass from the relatively warmer droplet edges are transferred along the surface towards to center where subduction occurs inwardly and solvation of the enriched polymer can further reduce free energy [11,12,13]. 

In each of these cases, surface perturbations directly respond to the moderately-unstable physical state of the interface and correspond to spatial wavelengths of the locally structured surface wrinkles. Spatial wavelengths that describe local surface periodicity tend to a critical value that corresponds to the critical nondimensional number for the observed instability. *Ra* describes the rate of thermal transport by diffusion with respect to buoyancy driven convection. *Ra* is critical when thermal transport is equal. Above critical *Ra*, the surface is destabilized by buoyancy and convective cells form at the surface. At the critical value of *Ma*, an instability causes the surface to roughen and the free energy to decrease. The sign for $\frac{\partial \sigma }{\partial T}$ is based on whether the surface is stabilized or destabilized by evaporation [14,15,16].

Buoyancy is a body force and by connection through density, it scales by volume. Surface energy scales linearly with the vertical height of the fluid. In a typical fluid environment, surface tension plays a passive role in the transport process. When the fluid height is sufficiently small, scaling laws are negligible, and interactions at the free surface can become the dominant factor. Because the surface tension of a solvent such as toluene (28.52 mN/m) is lower than that of PMMA (41 mN/m) [17], $\frac{\partial \sigma }{\partial T}$ is positive and evaporation causes the free energy of the surface to increase. *Ma* decreases with evaporation rate and fluid thickness. Because evaporation rate is scaled by the square of spin speed, *Ma* is at a maximum value when the fluid is stationary. 

A reduction in PMMA surface wrinkling may be possible by targeting fluid states below instability thresholds and at non-equilibrium transitions between dominant convection modes. For either instability to roughen the surface, thermal transport by convection must be greater than by diffusion alone. Defect free films should be producible at low film thicknesses, which are below the threshold conditions for an instability to form. A secondary interfacial instability may evolve at the transition between surface tension and buoyancy driven instabilities. If a moderately-unstable free energy state is formed at the transition between surface tension dominated and buoyancy dominated instabilities, two instabilities may converge and produce a secondary instability of lower free energy [18].

**Scheme 2:**
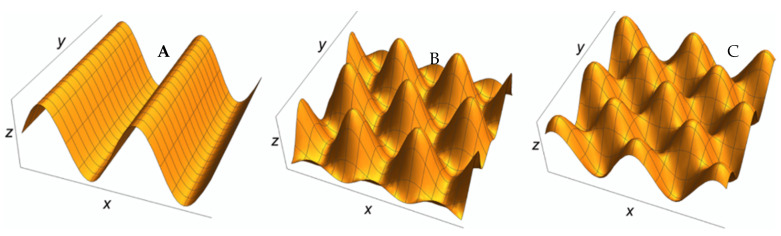
Surface plots of commonly observed arrangements of convective flow cells. (**A**) Rolls, (**B**) hexagonal cells, (**C**) square cells.

The geometry of surface wrinkling appears similar to oscillating wave vectors. Scheme 2 shows surface plots for three commonly observed arrangements of flow cells. Interfacial rolls, or wrinkles, are associated with buoyancy driven convection and approximate a sinusoidal profile that can be described by Equation (3).

(3) $$h\left(x\right)=h_{0}+a_{0}\sin\left(\frac{2\pi x}{\lambda_{0}}\right)$$

where $h_{0}$ is the mean thickness throughout the extracted region, $a_{0}$ is the wrinkle amplitude, and $\lambda_{0}$ is the wrinkle wavelength. Hexagonal cells have been considered the most stable morphology of primary instabilities [18] and tend to a triangular super-positioning of three wave vectors that can be described by Equation (4):

(4) $$h\left(x\right)=h_{0}+a_{0}\left[2\cos\left(\frac{\sqrt{3}}{2}\frac{x}{\lambda_{0}}\right)\cos\left(\frac{1}{2}\frac{y}{\lambda_{0}}\right)+\cos\left(\frac{x}{\lambda_{0}}\right)\right]$$

Secondary instabilities at non-equilibrium transitions can assemble as the super-positioning of convection rolls vectors that can be described by Equation (5) [13,15,19,20,21,22]:

(5) $$h\left(x\right)=h_{0}+a_{0}\left[2\cos\left(\frac{1}{\sqrt{2}}\frac{x}{\lambda_{0}}\right)\cos\left(\frac{1}{\sqrt{2}}\frac{y}{\lambda_{0}}\right)\right]$$

In this paper, we compare our theoretical results with what we observe experimentally using phase shifted interferometry. First, a qualitative assessment of PMMA film surface morphology at the spinning center is made. Then, interfacial roughness is characterized by dimension and shape, the parameters for spin-coating flatter films are realized, and relationships between instability flows and roughness morphology are inferred.

## 2. Materials and Methods

Glass microscope slides (~1 mm thick) were cut into 37.5 × 25 mm^2^ sections. Slides were cleaned and treated by submersion in ethanol (EtOH, 95%, Pharmaco-Aaper, Brookfield, CT, USA), followed by 15 min of sonication, then rinsed with purified water, submerged in purified water, sonicated for an added 15 min, and dried under N_2_.

PMMA (M_w_ ~120,000) was obtained from Sigma-Aldrich (Milwaukee, WI, USA) and used without further purification. A 20 g/dL stock solution was prepared in toluene (Honeywell, Charlotte, NC, USA, HPLC grade). The polymer stock solution was sonicated for 8 h with intermittent shaking to ensure full solvation. The resulting solution was optically clear. Dilutions ranging from 0.75 to 8.00 g/dL were prepared directly from the stock.

Sub-micron PMMA films were applied to the glass slides in dry conditions (<20% Relative Humidity) by depositing a 250 µL aliquot of polymer solution to the center of a slide and then spin-coating for 45 s at an acceleration of 1080 s^−2^ until reaching a maximum rotation speed between 400 and 8000 rpm. Films were air dried for 15 min and then transferred to an oven set at 60 °C for 2 min to facilitate residual solvent removal. Film thicknesses ranged from 75 to 700 nm.

Reflection spectra of the polymer films were acquired with a Filmetrics (San Diego, CA, USA) F40 microscope thin film analyzer. Reflectance in the range of 400–900 nm was generated by a tungsten-halogen light source. PMMA thin film optical constants for the refractive index and attenuation coefficient were obtained from literature [23]. Spectral fitting and film thickness calculations were performed with Filmeasure software. Transmittance spectra of polymer films were collected with a Perkin Elmer (Waltham, MA, USA) Lambda 1050 UV/Vis spectrometer. The slit width and integration time were set at 2 nm and 0.20 s, respectively. Wavelengths generated from tungsten-halogen and deuterium lamps were collected in the 1100 to 300 nm range. Optical micrographs and Three-dimensional surface profiles of dried polymer films were collected with a Filmetrics (San Diego, CA, USA) Profilm3D optical interferometer and then digitally extracted by instrument software. 

## 3. Results and Discussion

**Figure 1:**
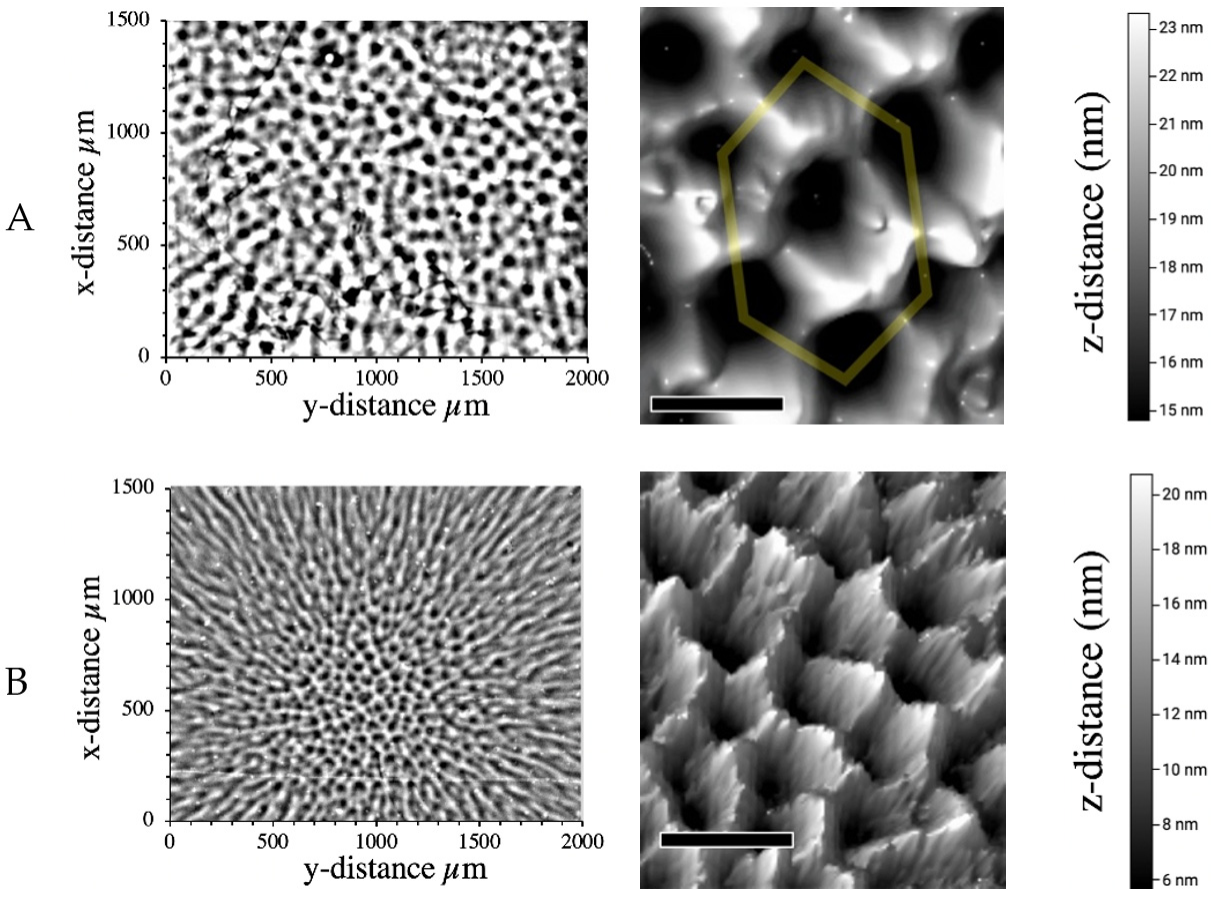

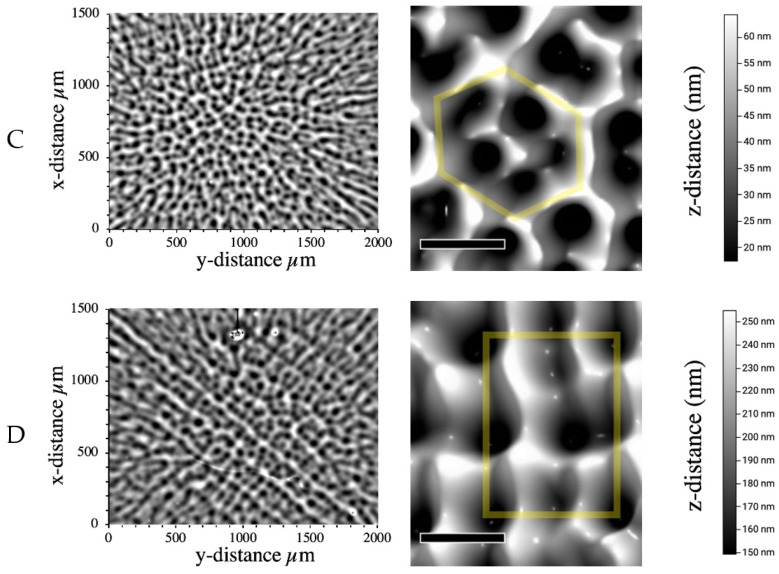
Optical micrographs showing surface morphology at the spinning center of PMMA films prepared from various concentrations and spin speeds. (**A**) Surface profile of 290 nm thick PMMA film cast from a 2.5 g/dL solution at 400 RPM. (**B**) Surface profile of 105 nm thick PMMA film cast from a 2.5 g/dL solution at 3000 RPM. (**C**) Surface profile of 305 nm thick PMMA film cast from a 4.0 g/dL solution at 1200 RPM. (**D**) Surface profile of 550 nm thick PMMA film cast from a 6.0 g/dL solution at 1200 RPM. Micrographs on the left are large area, 1500 × 2000 mm^2^ while the micrographs on the right are 310 × 275 nm^2^. The scalebar for the micrographs on the right is 100 nm. The gray-scale z-scale on the right applies to both micrograph magnifications.

A qualitative assessment of PMMA surface wrinkling was made by examining dry films near the spinning center. Figure 1 shows two different magnifications of micrographs for four PMMA films that were spin-cast from different solution concentrations and spin speeds. The lower magnification micrographs provide a view of global surface structure where disordered polygons with local periodicity are clustered around the spinning centers. As distance from the rotor position is increased, the shape of the surface wrinkling transitions from polygons to radiating rolls. The spatial dimensions of polygons and rolls average in the tens of microns for wavelengths and tens of nanometers for amplitudes. The higher magnification micrographs highlight local regions where unit-cells resemble characteristic geometries of common flow cell arrangements.

Figure 1A shows a film generated from parameters 2.5 g/dL and 400 RPM. The lower magnification view shows a disordered arrangement of round depressions. At higher magnification there is some local structuring of hexagons present. This type of inward flow is associated with surface tension driven instabilities [12]. Figure 1B shows how the surface changes when drying rate is increased by ~2.7 times. The lower magnification shows that the transition between rolls and polygons occurs at a closer distance to the spinning center. The higher magnification shows sharper peaks with smaller amplitudes and wavelengths. No local unit cells are seen. The higher disorganization at the increased drying rate is expected as increased air flow is known to promote surface defects [24]. Figure 1C shows a film generated from parameters 4.0 g/dL and 1200 RPM. Disorganized clusters of hexagonal flow cells that subdivide into five cells of shallower roughness can be seen at both magnifications. Figure 1D shows that when spin speed is held constant and polymer concentration is increased by 2 g/dL, the polygon clusters change from hexagons to squares. Square flow cells were the least common geometry observed at the spinning center. Square cells were mainly found on films cast at 1200 RPM and sometimes appeared together with large hexagons on PMMA films thicker than 550 nm. Square flow cells appear to have an equal inward and outward flow. They have been associated as a secondary instability of buoyancy and surface tension. Non-equilibrium phase transitions between moderately-unstable modes can be triggered by internal fluid motion [25].

**Figure 2:**
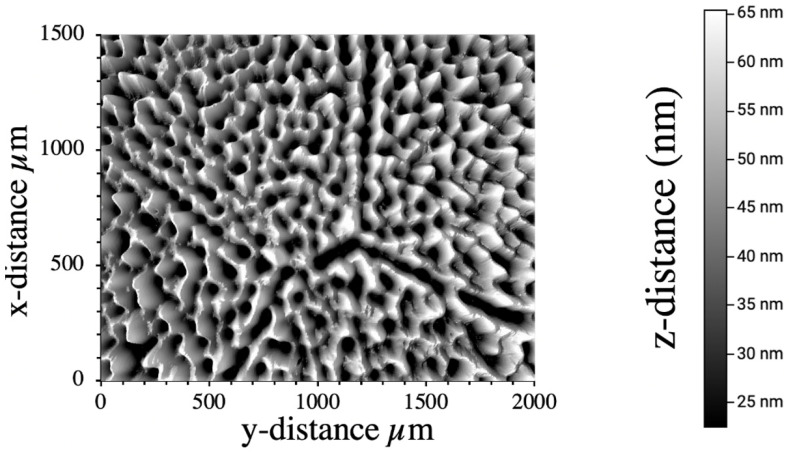
Optical micrograph showing surface morphology at the spinning center of PMMA film produced from 8.0 g/dL and 1000 RPM.

Figure 2 shows a centrally positioned digital surface profile with both inwardly and outwardly flowing hexagons. Most of the surface roughness consists of inwardly flowing disorganized polygons that look similar to surface of the fast-drying film in Figure 1B. A second and much larger hexagon appears superimposed and flowing in the opposite direction. Films cast from concentrated solutions, such as 8.0 g/dL, have segments of large hexagonal cells which more closely resemble the whale oil convection cells of Bénard [6]. The superimposed hexagons may suggest a non-equilibrium dual-instability mode with large buoyancy driven wavelengths and small surface tension dominated wavelengths. This type of dual-instability surface wrinkling was only found on PMMA films thicker than 360 nm.

**Figure 3:**
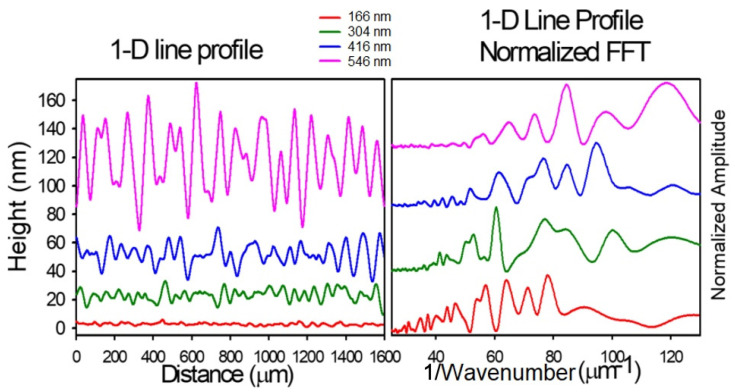
One-dimensional line profiles of PMMA surfaces at locations where convection rolls are present. (**Left**) PMMA surface 1D line profiles. (**Right**) Height normalized Fast Fourier Transforms of the corresponding line profiles. Films were cast using 2.5, 4.0, 5.0, and 6.0 g/dL solutions spun at 1200 RPM.

Figure 3 shows one-dimensional line profiles of PMMA thin films generated from different solution concentrations at a constant spin speed. Each of the line profiles were taken at a distance of ~7.50 mm from the spinning center, which is in the region of convection rolls (wrinkles) for each of the films. Figure 3 also shows a height normalized fast Fourier transform (FFT) plot of the line profiles. Film thickness and the wrinkle amplitude both increase with casting solution concentration. From the FFT plot, the wavelength spacing of surface wrinkles is shown to increase with concentration, but to a lesser degree than wrinkle amplitude. Local segments of line profiles were discretely fit to Equation (3). Examples of the fits are shown in the Supplementary Materials (Figures S1 and S2).

A series of PMMA films were spin-cast using speeds between 400 to 8000 RPM and solution concentrations from 2.5–8.0 g/dL. Wrinkle amplitude and wavelength measurements were generated from one-dimensional surface profile sinusoidal fittings. A second set of measurements was made from two-dimensional parameters root-mean-square (RMS) roughness and autocorrelation length (ACL).

**Figure 4:**
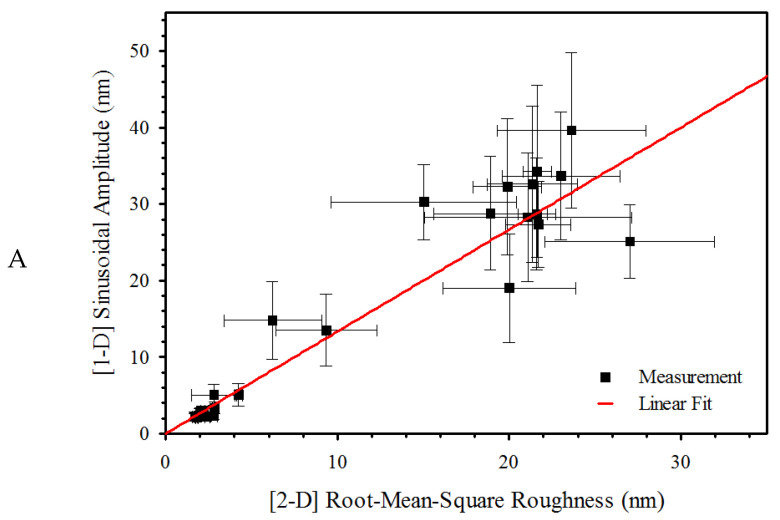

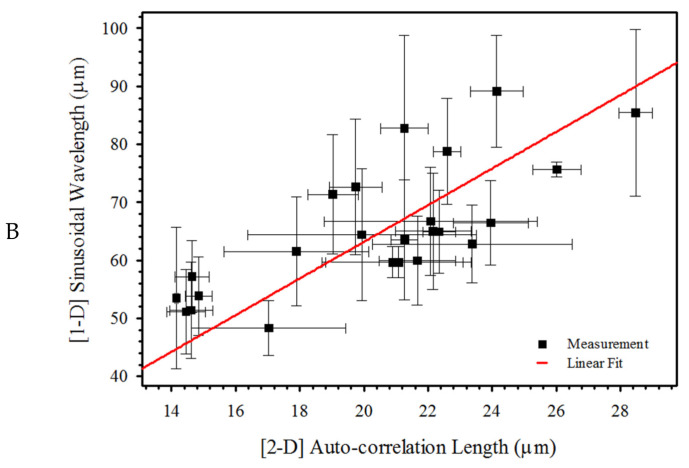
PMMA wrinkle measurements by one- and two-dimensional analysis methods. (**A**) PMMA surface wrinkle amplitude made by one-dimensional sinusoidal fits plotted versus two-dimensional RMS roughness. The slope and standard error are 1.34 ± 0.06. (**B**) Wrinkle wavelength made by one-dimensional sinusoidal fits plotted versus two-dimensional ACL. The slope and standard error are 3.16 ± 0.08. Vertical and horizontal error bars represent a standard deviation of at least ten sinusoidal fits and four RMS or ACL measurements.

Figure 4 shows the relationship between one- and two-dimensional measurements. The fits suggest that one- and two-dimensional measurement techniques may be related, as approximated in Equations (6) and (7).

(6) $$a_{0}\approx \;RMS\times \sqrt{2}$$

(7) $$\lambda_{0}\approx \;ACL\times \pi$$

These equations are phenomenological and we are not aware of any fundamental explanation.

**Figure 5:**
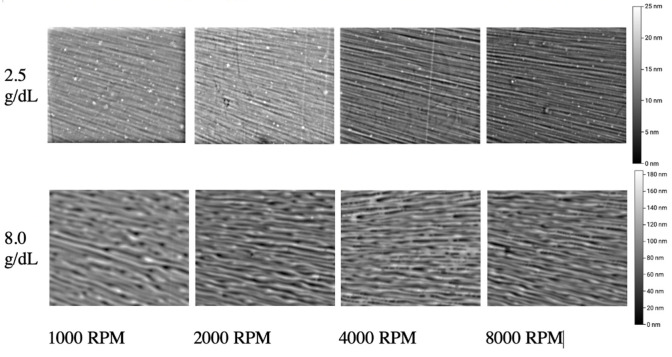
Optical micrographs of PMMA thin films cast at different spin speeds from chosen solution concentrations. The micrographs are taken at least 4 mm away from the center of the film where the wrinkle patterns are well developed.

As we reported with polystyrene, higher spin speed led to more uniform wavelengths and lower spin speeds produce a larger range of wavelengths [26]. This trend can also be seen in PMMA films, as shown in Figure 5. As spin speed increases, the wrinkle wavelengths appear more.

Films were prepared from solutions ranging in concentration from 2.5 g/dL to 8.0 g/dL and spin speeds from 1000 RPM to 8000 RPM. This gave film thicknesses ranging from ~100 nm to ~800 nm but for a given thickness there could be multiple concentration/rotation rate combinations. All of these films formed periodic surface structures 3 mm and greater from the spinning center. The structures were fit using Equation (3) (examples are given in Supplementary Materials). The aspect ratios (width/depth) of these interfacial wrinkle structures are in the thousands. Similar aspect ratios observed with polystyrene thin films cast from binary solvents THF and DMF [26].

**Figure 6:**
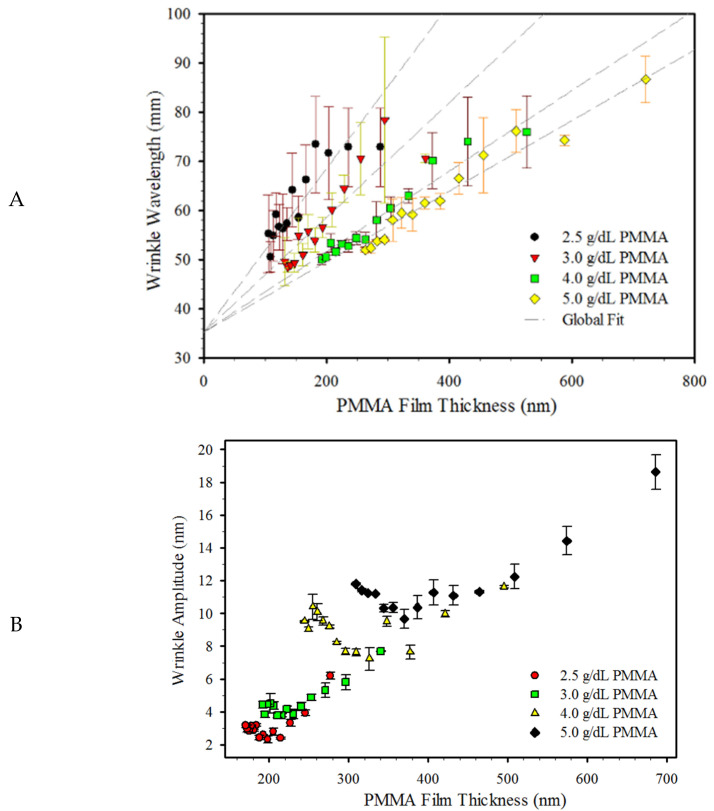
PMMA surface wrinkle wavelength (**A**) and wrinkle amplitude (**B**) as a function of final film thickness. PMMA thin films were cast from 2.5, 3.0, 4.0, and 5.0 g/dL solutions spun at speeds from 400 to 3000 RPM. The error bars represent the standard deviation of at least four different determinations taken at increments of 3.75, 7.50, 11.25, and 15.00 mm from the center of each film.

Figure 6 shows wrinkle wavelengths as a function PMMA thicknesses grouped by casting solution concentration. Wrinkled surfaces with local periodicity were seen on the surfaces of films with thicknesses larger than 75 nm. A critical minimum wrinkle wavelength of ~45 μm is observed on the thinnest films. Linear correlation between wrinkle wavelength and average film thickness has been previously identified for thicker films [10,16,27]. When the data set for each concentration were fit to a linear equation it appeared that there was a common intercept. Thus, the wavelength data for each concentration (*λ_o_*(*conc*)) in Figure 6A were globally fit to the equation *λ*_o_(*conc*) = *λ*_univ_ + *m*(*conc*)*h*_f_, where *m(conc)* is the concentration dependent slope and *λ*_univ_ is the shared parameter for the wavelength-intercept. The slope of each fit increases with decreasing casting solution concentration. The shared intercept was found at 35.3 ± 1.4 μm (shown in Table S1). To the best of our knowledge, a common intercept for instability wavelength has not been published. The linearity of the wrinkle wavelengths as a function of film thickness implies that the dominant force driving the periodic structure is the Marangoni effect, as suggested by Equation (2). 

Figure 6B shows a plot of wrinkle amplitude as a function of average film thickness. For films cast from a constant concentration, wrinkle amplitude has a nonlinear relationship with respect to film thickness. For thin films the average wrinkle amplitude decreases with increasing film thickness, reach a minimum, and then increase approximately linearly with increasing film thickness. This behavior implies a complicated balance between buoyancy and surface tension effects. 

**Figure 7:**
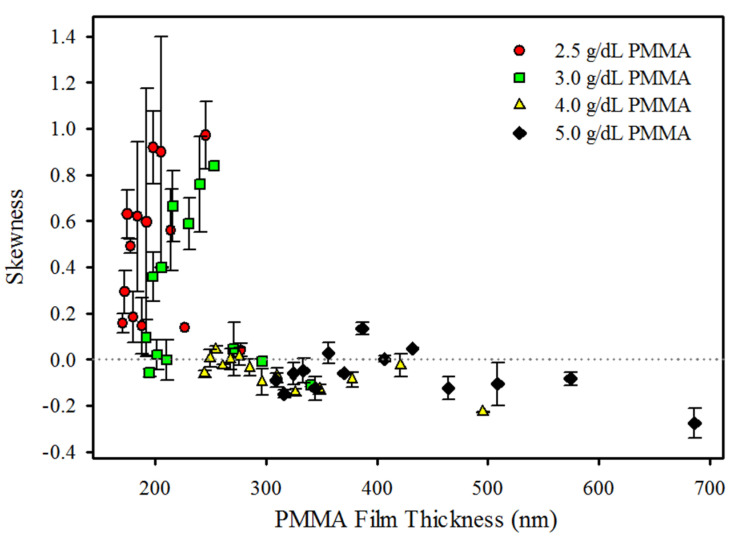
PMMA surface skewness as a function of final film thickness. Films were cast using 2.5, 3.0, 4.0, and 5.0 g/dL solutions spun at speeds from 400 to 3000 RPM.

Figure 7 shows the skewness of the wrinkles compared to the thickness of dry PMMA films. A horizontal dashed line has been drawn at zero skewness, where an even distribution of wrinkle peaks and valleys would lie. The error bars represent the standard deviation of four different skewness measurements collected at increments of 3.75, 7.50, 11.25, and 15.00 mm from the center of each film. The surface of some PMMA thin films with thicknesses between 170 and 250 nm show positive skew, indicating wrinkles with sharp peaks and shallow valleys. PMMA films between 250 and 500 nm have vertically symmetrical wrinkles with a Gaussian character. This change in skewness as a function of casting solution concentration may be related to the hydrodynamic radius of the polymer existing when the film was vitrified. A possible explanation for the clear cutoff in skewness at ~250 may suggest a casting solution concentration threshold for globular folding/unfolding. The changing wrinkle skewness at lower thicknesses may also be influenced by shear-flow effects from spin speed [28].

The total drying rate during spin-coating is related to solvent static evaporation rate ***e*** and the square root of spin speed (ω) [29,30]. Therefore, the drying rate can be calculated from Equation (8).

(8) $$E=e\omega^{1/2}$$

**Figure 8:**
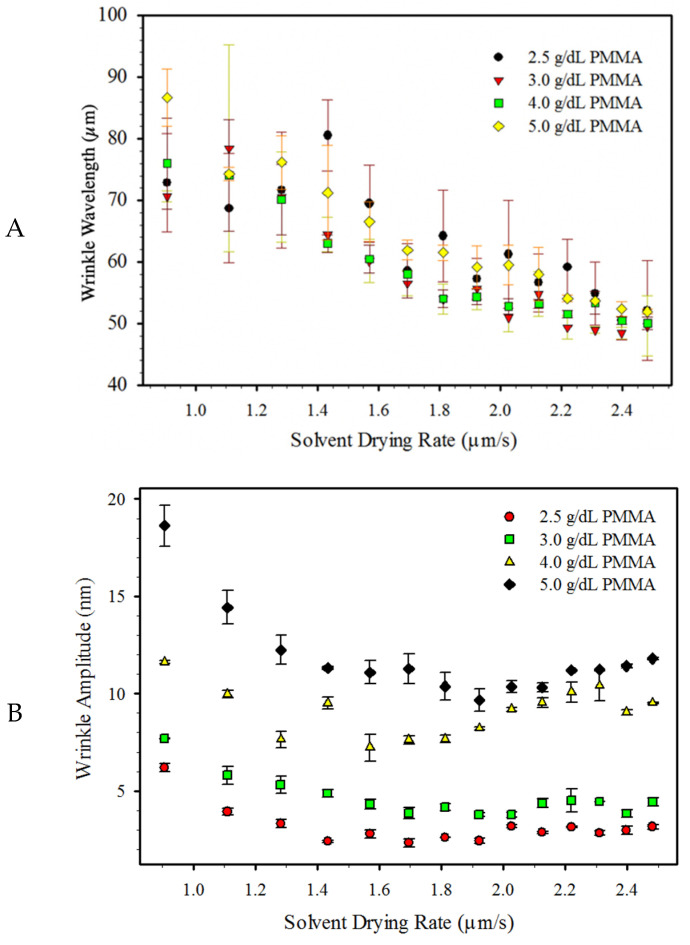
PMMA surface wrinkle wavelength (**A**) and wrinkle amplitude (**B**) as a function of solvent drying rate. Films were cast using 2.5, 3.0, 4.0, and 5.0 g/dL solutions spun at speeds from 400 to 3000 RPM. The error bars represent the standard deviation of four different ACL and RMS roughness measurements taken at incremental distances of 3.75, 7.50, 11.25, and 15.00 mm away from the spinning center.

The static evaporation rate for toluene was calibrated with the film thickness model and found to be $\sqrt{\omega }\times$140 nm/s^1/2^ [30]. The link between surface structures and drying rate has been established [31,32,33]. The effect of solvent drying rate on wrinkle wavelength is shown in Figure 8A. When the drying rate is 0.9 µm/s, the average wrinkle wavelength is between 65 and 90 µm for films cast from each of the concentrations. As the drying rate is increased to 2.5 µm/s, the wrinkle wavelength decreases to ~52 µm. As seen in Figure 8B, the spin-casting solution concentration appears to affect wrinkle amplitude, but not wavelength. At the same drying rate, films cast from higher solution concentrations form wrinkles with larger amplitude. 

Since film height is scaled by the inverse square root of spin speed, smaller films are formed with faster drying rates and larger temperature gradients [27]. As surface tension driven instabilities decrease, and buoyancy driven instabilities increase with growth of temperature-gradients, film thickness increases. The relationships near 1.6 µm/s shown in Figure 6B and Figure 8B may describe non-equilibrium transitions between dominant convection modes.

## 4. Conclusions

The surface morphology of spin-cast PMMA films have been examined using phase-shifted interferometry. The experimental results demonstrate that roughness can be modulated from casting solution concentration and solvent drying rate. Local regions with hexagon and square unit cells were observed near the spinning centers, but a long-range periodic structure was lacking. Relatively flat surfaces were found on films with thicknesses below 75 nm. Interfacial wrinkles can be minimized by targeting solvent drying rates between 1.4 to 1.7 µm/s. A deeper understanding of fluid flows and surface tension phenomenon has been attained. Insights into the spin-coating process were gained, such as faster drying rates on the thinner films results in rougher surfaces, wrinkle amplitude can increase or decrease with film thickness, and that average wrinkle wavelength decreases with increasing drying rate. A critical wavelength for roughening of the surface by convection instabilities was observed at around 45 microns.

## Data Availability

The data presented in this study are available on request from the corresponding author.

## Supplementary Materials

The following are available online at https://www.mdpi.com/article/10.3390/polym13132184/s1, Figure S1: Line scans and fits for several PMMA surfaces cast from a 2.5 g/L solution, Figure S2: Line scans and fits for several PMMA surfaces cast at 2000 RPM, Table S1: Fit parameters for Figure 6A.
