# Supplementary material for: Evolution of Surface Morphology of Spin-Coated Poly(Methyl Methacrylate) Thin Films
Source: Polymers (Basel). 2021 Jun 30;13(13):2184. doi: 10.3390/polym13132184 (PMC8271514; doi:10.3390/polym13132184)
Supplement: Supplementary file 1 [file polymers-13-02184-s001.zip › polymers-1286200-supplementary.pdf]

Evolution of Surface Morphology of Spin-Coated Poly(methylmethacrylate) Thin Films

Navid Chapman, Mingyu Chapman, and William B. Euler*

Department of Chemistry, University of Rhode Island, 140 Flagg Road, Kingston, RI, 02881

Supplementary Materials

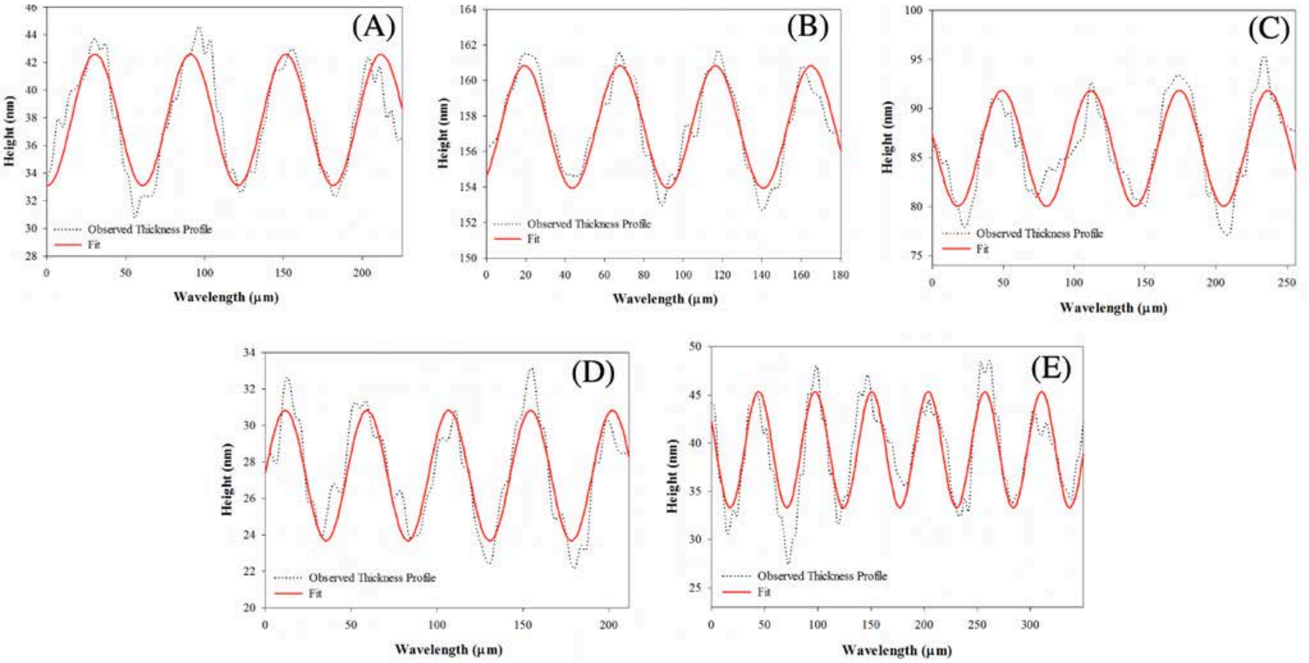

Figure S1. Line scan profiles of the wrinkled PMMA surfaces for films cast from 2.5 g/dL. Black lines are the observed profile data and the red lines are fits. (A) ω = 2000 RPM, a_0 = 4.7 ± 0.3 nm, λ_0 = 60.4 ± 0.5 µm; (B) ω = 3000 RPM, a_0 = 3.5 ± 0.2 nm, λ_0 = 48.6 ± 0.3 µm; (C) ω = 5000 RPM, a_0 = 5.9 ± 0.3 nm, λ_0 = 62.5 ± 0.4 µm; (D) ω = 7000 RPM, a_0 = 3.6 ± 0.2 nm, λ_0 = 47.6 ± 0.4 µm; (E) ω = 8000 RPM, a_0 = 6.0 ± 0.3 nm, λ_0 = 53.3 ± 0.2 µm. Uncertainties are the standard error of the estimated fit.

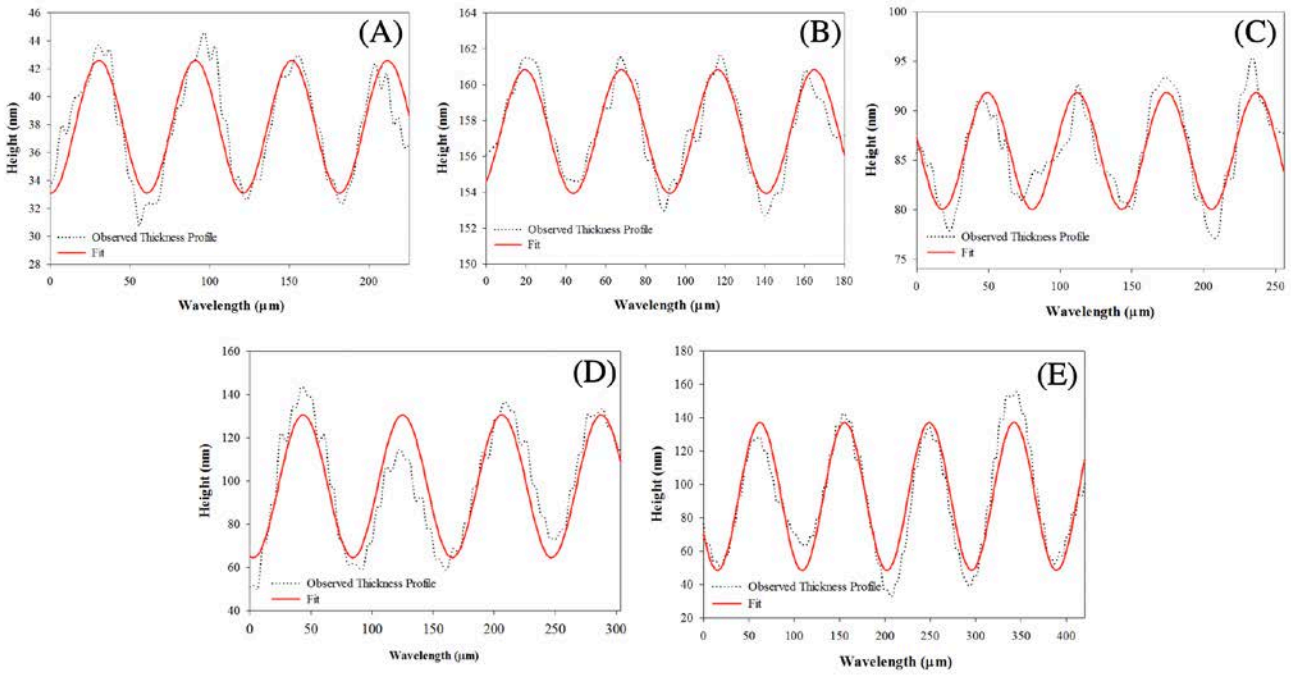

Figure S2. Line scan profiles of the wrinkled PMMA surfaces for films cast at 2000 RPM. Black lines are the observed profile data and the red lines are fits. (A) 3 g/dL PMMA, a_0 = 22.2 ± 0.9 nm, λ_0 = 74.8 ± 0.4 µm; (B) 4 g/dL PMMA, a_0 = 19.6 ± 0.9 nm, λ_0 = 76.1 ± 0.3 µm; (C) 5 g/dL PMMA, a_0 = 34.3 ± 1.4 nm, λ_0 = 68.9 ± 0.3 µm; (D) 6 g/dL PMMA, a_0 = 33.0 ± 1.7 nm, λ_0 = 81.4 ± 0.6 µm; (E) 7 g/dL PMMA, a_0 = 44.3 ± 1.4 nm, λ_0 = 93.4 ± 0.3 µm. Uncertainties are the standard error of the estimated fit.

Table S1. Fit parameters for the global fit from figure 6A. The shared intercept was 35.3 ± 1.4 µm.

**Concentration g/dL**	**Slope + error**	**r^2**
2.5	0.167 ± 0.010	0.69
3.0	0.117 ± 0.008	0.83
4.0	0.082 ± 0.005	0.95
5.0	0.072 ± 0.004	0.95
Overall Global Fit		0.87
